# Residential characteristics as correlates of occupants’ health in the greater Accra region, Ghana

**DOI:** 10.1186/1471-2458-14-244

**Published:** 2014-03-11

**Authors:** Emilia Asuquo Udofia, Alfred E Yawson, Kwesi Adu Aduful, Francis Mulekya Bwambale

**Affiliations:** 1 Department of Biological, Occupational and Environmental Health, School of Public Health, University of Ghana, Legon, Accra, Ghana; 2 Department of Community Health, University of Ghana Medical School, College of Health Sciences, Korle Bu, Accra, Ghana; 3 Department of Surgery, Komfo Anokye Teaching Hospital, Kumasi, Ghana; 4 School of Public Health, Makerere University College of Health Sciences, Kampala, Uganda

**Keywords:** Housing, Health, Built environment, Ghana

## Abstract

**Background:**

Housing has been a relatively neglected site for public health action. However, it remains a place where human beings spend the most part of their day. As a result, the quality of housing has consequences for human health. We investigate residential characteristics associated with self-rated occupant health in five neighbourhoods in the Greater Accra Region, Ghana.

**Methods:**

A cross sectional study using a semi-structured questionnaire was conducted among 500 informed adults aged 18 years and above to investigate residential characteristics associated with self-rated occupant health in five neighbourhoods in the Greater Accra Region, Ghana. Correlates of occupant rated health were determined using Pearson chi-square test and binary logistic regression.

**Results:**

Forty-two per cent of houses were rented, 44% required repair and 46% shared sanitation facilities. One in twenty occupants reported poor health. Gender, employment status, income, ventilation, house wall material, odours, stale air, privacy, shared facilities, hand washing facility, type of house and house repair status were associated with poor health in the bivariate analysis. Only two variables were independently associated with poor self-rated health: occupants who lacked privacy were eight times more likely to report poor self-rated health when compared to peers who did not lack privacy [OR = 8.16, 95% CI 2.86-23.26] and women were three times more likely than men to report poor health [OR = 2.98, 95% CI 1.06-8.35].

**Conclusion:**

The results provide further evidence of housing as a determinant of occupants’ health, and identify housing characteristics and living conditions as issues for public health action in Ghana.

## Background

“*We give shape to our buildings, and they in turn shape us.”*

*(Winston Churchill in a 1943 speech to the House of Commons) *[1].

The World Health Organization (WHO) states that the housing characteristics, community and neighbourhood environment have the potential to affect human health, through physical, mental and social mechanisms [2]. Adequate housing should provide shelter from climatic conditions, intrusions by vectors and rodents as well as environmental nuisances such as noise. It should also offer security and privacy [3]. Access to safe water and basic sanitation are critical to maintaining a healthy residential and neighbourhood environment. Studies on housing from developing countries have suggested that the provision of basic amenities may result in reduced illness [4]. Substandard housing has been associated with a diversity of health conditions including asthma, tuberculosis, lead poisoning, injuries and poor mental health [5]. It is in recognition of growing evidence regarding the association between housing and health that the United States Center for Disease Control and Prevention stresses the improvement of housing and living conditions as a strategy to promote health [4]. Most studies investigating the relationship between housing and health have been conducted in developed countries [6-11] while fewer studies have been reported in African countries [12-15], especially Ghana [13,16].

Although efforts are increasingly being made to improve housing standards and housing stock; population growth, urbanization, migration, natural disasters, conflicts and unemployment seemingly slow progress made in most developing countries. Standard housing can hardly be afforded by the urban poor who often resort to makeshift housing in insecure neighbourhoods where social amenities are scarce and environmental nuisances are commonplace. In Ghana, as in other developing countries such as Tanzania, Kenya and Nigeria, population growth and internal rural urban migration have contributed to the sprawl of unplanned informal settlements [13,17,18]. These settlements often have substandard housing characterized by poor structural quality, inadequate access to social amenities such as water and basic sanitation, insecurity of tenure and overcrowding [17].

It has been estimated that urban areas in Ghana will require nearly 2 million dwellings by 2020 if built as self-contained dwellings, one for each household. Currently about 90% of urban housing in Ghana is classified as informal due to their construction without local authority control and almost 60% of households occupy single rooms [19]. With the growth of cities, new rooms are added to existing houses in central areas. District hospital records indicate that respiratory illnesses and diarrhoea are among the top ten causes of outpatient hospital visits in Ghana, after malaria (unpublished: district hospital records compiled at the Department of Community Health, University of Ghana Medical School). Crowded housing is associated with higher rates of infectious disease transmission such as in respiratory infections and tuberculosis [5,13]. Lack of safe drinking water, ineffective waste disposal and inadequate food storage contribute to the transmission of diarrhoeal diseases.

The Urban Multiple Indicator Cluster Survey (MICS 2011) conducted among residents of Accra living in five high density urban neighbourhoods showed that only 11% of households were using an improved sanitation facility or toilet [20]. Fifty two per cent of households use public facilities and nearly 12% of households share the toilet facility among 5 or more households [20]. Studies indicate that health problems from lack of sanitation facilities are greater among residents of informal settlements and deprived poor communities compared to towns and cities in Ghana [20].

The house is where most individuals spend the most part of their day. For the employed working an eight hour job, this would translate to a maximum of 16 hours. For individuals who are at extremes of age (pre-schoolers and elderly), homemakers and pregnant women for instance, the time spent at home could be much longer. Previous studies have shown that the quality of the house has the potential to affect the health of its occupants through various exposures and the time spent within the exposures, although host immunity and body surface area are other important factors. This is well expressed in the “rule of 1000” which states that a pollutant released indoors is 1000 times more likely to reach people’s lungs than a pollutant released outdoors [21]. This present survey was conceived to obtain local evidence linking housing and health in five neighbourhoods with varying characteristics in the Greater Accra Region (GAR), as well as determine if the results would corroborate evidence from previous studies [13,14]. The results provide evidence of association with occupant health for some characteristics suggesting that improving housing quality has the potential to promote health in Ghana.

## Methods

### Study design and setting

**Figure 1:**
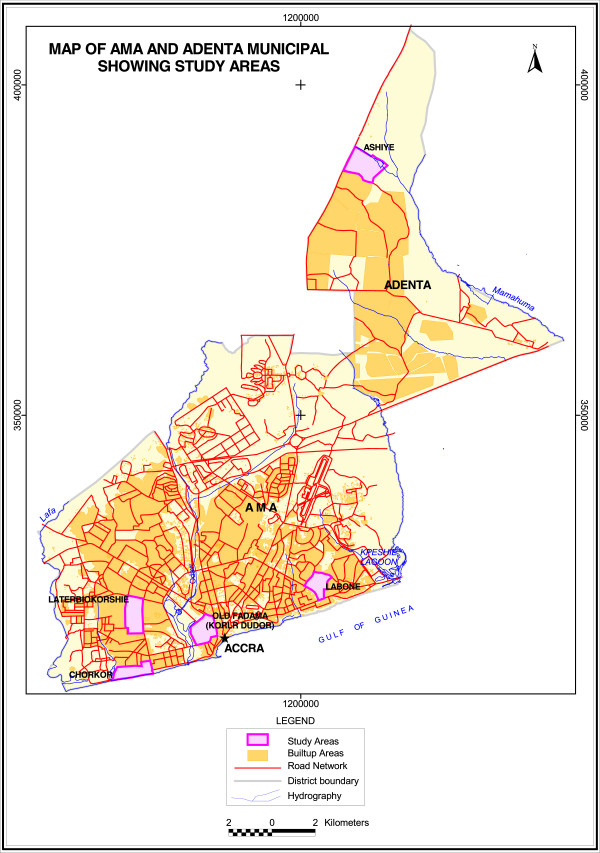
**Map showing the study areas.** A map of the study areas showing four neighbourhoods (Laterbiokoshie, Labone, Old Fadama and Chorkor) in the Accra Metropolitan Area (AMA) and one neighbourhood (Ashiyie) in Adenta Municipality.

A cross sectional study was undertaken as both risk factors and the outcome were determined simultaneously. The administrative structure of Ghana comprises regions, metropolitan assemblies, municipal assemblies and district assemblies. The metropolitan assemblies are further divided into sub-metropolis. Data collection took place in five purposively selected neighbourhoods (Figure 1) in the Greater Accra Region (GAR), Ghana, over the four weeks in August, 2012. The GAR has the second largest population in Ghana (4 010 054 people) and the largest proportion of urban dwellers [22]. The neighbourhoods were selected to reflect rural, peri-urban and urban settings in GAR with varying socio-demographic and environmental characteristics. Old Fadama located in Ashiedu Keteke Sub-metropolis also known as Korle Dudor, is Ghana’s largest slum and home to nearly 80,000 people accommodated on land reclaimed from the Korle Lagoon [20,23]. Residents are mainly traders and head porters at a major market, Agblogbloshie market. Many live in substandard housing and have faced several eviction threats. Chorkor is a traditional fishing community and one of the most densely populated communities in the Ablekuma South Sub-Metropolis with a population of 78,918 people. It lies within the poverty pockets of Accra, which are characterized by people who lack information, power and resources and often are excluded from development intervention [24].

Labone is situated in La Dade Kotopon Sub-metropolis and has a population of 183, 528 [22]. Houses in this middle to high income, urban neighbourhood are a mix of detached flats and storey buildings. Lartebiokorshie is a medium density urban settlement in the Ablekuma Central District. It has a mix of affluent residents in the Radio Gold and Bishop Bouwers divisions, while those in the lower socioeconomic class live along the Town Council line. All four locations described are under the Accra Metropolitan Assembly. Ashiyie is a rural town situated within Adenta Municipal Assembly, known as the Koose electoral area. It has an estimated population of about two thousand people (personal communication with Unit Secretary, Mr. Enoch). Estate development is ongoing in this area and engages most youth in construction activities.

### Ethical considerations

Ethical approval for this research was obtained from the Ethical and Protocol Review Committee of the University of Ghana Medical School. Permission to conduct the survey was given by the Assembly men and women of the respective study sites directly or conveyed through the Unit Secretaries. Permission to interview a respondent in the household was obtained from the household head or a responsible adult acting on his or her behalf. Individual informed consent was obtained from the respondents who were interviewed in their houses for convenience and to facilitate inspection where this was permitted by the occupant. The inspection was not conducted if the respondent declined. Anonymity was ensured by the use of codes and access to data was restricted to the researcher and interviewers only.

### Study population and sampling

The study population comprised of adults aged 18 years or older, living in a housing unit in any of five purposively selected neighbourhoods. A total sample of 500 informed (capable of providing information about the house and household) adults aged 18 years and older, living in a housing unit in the selected neighbourhoods, was enlisted for the study comparable to a previous survey by Arku et al. [13].

Cluster sampling method was used to reach eligible respondents. Given that some of the study sites were informal settlements (Chorkor, Old Fadama) and most of the study sites did not have numbered dwellings, preliminary visits were made by the researcher and interviewers to map out the study sites in clusters divided mainly by streets and specific landmarks. The clusters were assigned serial numbers and a ballot drawn by the research team to pick one cluster in each site. A total enumeration of housing units was conducted in the selected cluster. A housing unit was defined as the regular accommodation of the respondent where household activities take place and possessions are kept. In each study site, we aimed at reaching 100 respondents in order to capture a variety of living conditions and attain the final sample size. Where this number was not attained in any cluster, a ballot was drawn to pick another cluster to continue enumeration until at least 100 housing units were covered. Where more than one eligible adult was present in a house, a simple ballot was performed by the interviewers to select the respondent.

### Data collection

A 62-item, semi-structured questionnaire was administered to respondents by a team of four trained interviewers. The questionnaire had six major themes namely: socio-demographics, housing characteristics, amenities, hygiene, sanitation and refuse disposal, health and physical complaints. All questionnaires were administered in the language of preference to the respondents during weekends and in the afternoon and evenings during weekdays. Where permitted, an inspection of the house was performed to observe the presence of uncovered or spilled refuse, animal dander, and drains and observations were noted on a checklist.

### Study variables

The independent variables were: socio-demographic variables namely age, sex, marital status, religion, educational attainment, employment status and income; housing variables namely type of house, structural materials (house walls), occupancy, ventilation (number of windows per bedroom), daylight penetration, tenure, length of residency, and repair status; housing facilities namely electricity, water supply, availability of water supply, sanitary and hand washing facilities in premises, and refuse disposal system; and housing conditions namely presence of specific problems (dampness, dryness, dust, odours, indoor smoke, stale air, outdoor smoke), presence of pests, presence of animals and sanitation status. Privacy and fear of ejection were ascertained as specific questions. Both variables were used in a previous study as indicators of housing demand and control that could affect self-rated and mental health [13,14,16]. A question was also asked about common illnesses in the neighbourhood (malaria, typhoid, acute respiratory infection, skin infection, gastroenteritis and others). Under ‘others’, respondents were allowed suggest other diseases not included in the foregoing list. Respondents were asked if any member of the household had been ill with any of the diseases listed in the last six months. The recall period of six months was allowed to accommodate the possibility of occurrence of any of the illnesses listed. Although a shorter period may have been more effective for recall, such as two weeks, it is likely that some illnesses may not have occurred in that time frame. Ten specific physical complaints experienced at least once a month (headache, dizziness, watery eyes, itchy skin, cough, difficulty hearing, chest pain or tightness, watery stools, abdominal pain and spiking temperature) were also listed and respondents asked if they had experienced any of these. While the first seven physical complaints are consistent with those reported in literature associated with poor housing, the latter three non-specific complaints were added as dummy options.

For inferential analysis, all explanatory variables were dichotomized such that the risk status was represented by ‘1’ and the health promoting status was represented by ‘0’.

The dependent variable was self–rated (occupant health) which was measured by asking the following questions: “*Compared to other people your age, how would you assess your general health*?” The options provided were in ranked order: (1) excellent, (2) very good, (3) good, (4) fair and (5) poor. In the binary logistic analysis, the categories (1), (2) and (3) were merged as good health, while (4) and (5) were merged as poor health as consistent with a previous study [13]. Therefore the final outcome variable was dichotomous with two levels namely ‘good health’ and ‘poor health’.

### Data analysis

The data were entered into an electronic database and analysed using Statistical Package for Social Sciences (SPSS) version 17. Descriptive statistics for categorical and continuous variables were summarised using frequency distributions and percentages for categorical variables; and the mean, standard deviation, mode, maximum and minimum values for continuous variables. Pearson chi square test was used to test for the association between independent variables and occupants’ health. Fisher’s exact test was used for any cell with smaller than 10 counts. A p-value of 0.05 or less was deemed statistically significant. Only variables statistically significant in the bivariate analysis at the p-value of 0.5 or less were evaluated with binary logistic regression using the backward elimination method. The binary logistic regression analysis was used as the dependent and explanatory variables were dichotomous. The variables in the final model included: type of house, house wall material, shared toilet, hand washing facility, number of windows per bedroom, sex, employment status, household income, stale air present, odour present, house needs repair, has privacy in the house, and location. These were regressed against self-reported ill health. The strength of association was determined by the adjusted Odd’s ratio (OR) and the 95% confidence limits were constructed around the estimates.

## Results

### Socio-demographics

The socio-demographic characteristics of the study participants are shown in Table 1. The modal age group was found among respondents aged 26–35 years (29.7%; n = 148) followed by 36–45 years (27.7%; n = 138). The sex distribution was nearly equal with females constituting 49.4% (n = 247). Nearly half of the residents were married (49.8%; n = 249) and a third (33.8%; n = 169) were single. One hundred and seventy two (34.4%) respondents attained tertiary level education or higher, while 29(5.8%) had no formal education. The majority of respondents were either in government (20.4%; n = 102) or private employment (18.0%; n = 90). The modal household income level was GHC 100–500 (USD 50 – 250) accounting for 231 (46.4%) respondents. Minimum daily wage in Ghana at time of data collection was GHC 3.73 (i.e. GHC 112 per month; USD 56 per month). More than 10% of households surveyed earned less than the national minimum daily wage.

**Table 1:** Socio-demographic characteristics of study participants, Greater Accra Region, Ghana, 2012

**Variable**	**Self-rated health**	
	**Good health**	**Poor health**
Age in years (n = 499)	Frequency (%)	Frequency (%)
18-25	100 (94.3)	6 (5.7)
26-35	140 (94.6)	8 (5.4)
36-45	133 (96.4)	5 (3.6)
46+	101 (94.4)	6 (5.6)
Gender (n = 500)		
Male	245 (96.8)	8 (3.2)
Female	229 (92.7)	18 (7.3)
Marital status (n = 500)		
Never married	157 (92.9)	12 (7.1)
Married	239 (96.0)	10 (4.0)
Co-habiting	17 (89.5)	2 (10.5)
Divorced/separated/widowed	61 (96.8)	2 (3.2)
Educational status (n = 500)		
No formal education	27 (93.1)	2 (6.9)
Primary/basic/other*	64 (91.4)	6 (8.6)
Senior secondary	119 (90.8)	12 (9.2)
Vocational/technical	94 (95.9)	4 (4.1)
College/tertiary	170 (98.8)	2 (1.2)
Occupation (n = 500)		
Unemployed	41 (91.1)	4 (8.9)
Student	66 (95.7)	3 (4.3)
Home maker	22 (95.7)	1 (4.3)
Farming	17 (98.5)	2 (10.5)
Petty trading	69 (88.5)	9 (11.5)
Artisan	71 (95.9)	3 (4.1)
Government employed	101 (99.0)	1 (1.0)
Privately employed	87 (96.7)	3 (3.3)
Household income (n = 498)		
<100 GHC	46 (90.2)	5 (9.8)
101-500 GHC	220 (95.2)	11 (4.8)
>500 GHC	100 (99.0)	1 (1.0)
Not applicable	106 (92.2)	9 (7.8)

*Other = Islamic and other schools at the basic level; all (%) refer to row percentages.

### Housing characteristics

The housing characteristics and living conditions of the study participants are shown in Table 2, while the numerical parameters (age, room occupancy, rooms and persons per house) are shown in Table 3. Two hundred and eight respondents (41.7%) lived in rented accommodation and a quarter of the respondents (25.4%; n = 126) considered their rent affordable. Four hundred and thirty eight (88.7%) respondents lived in their homes longer than six months with 160 (32.1%) having lived in houses that were more than 10 years old. Three hundred and fourteen (62.8%) respondents lived in self-contained houses and single family detached houses. Most houses were made of cement, 386 (77.2%) had aluminium roofing sheets, 294 (58.8%). At a crowding threshold of 2 persons per room, 184 (36.8%) houses were overcrowded. Houses had an average of: 7 persons per house (6.91 +/- 6.25), 4 rooms (4.37 +/–3.14) and 2 persons per room (2.43 +/–1.55). However it must be noted that the distributions of persons per house and rooms per house were positively skewed which will affect the mean values. The respective median values were 5 persons per house and 4 rooms per house. The skewed distribution was due to outliers which were not excluded during analysis because these were found to be concentrated mainly in compound houses, a plausible explanation in spite of the extreme values.

**Table 2:** Housing characteristics and living conditions of the study participants, Greater Accra Region, Ghana, 2012

**Variable**	**Self-rated health**	
	**Good health Frequency (%)**	**Poor health Frequency (%)**
Physical structure (n = 500)		
Cement/concrete	367 (95.1)	19 (4.9)
Brick/mud/wood	107 (93.8)	7 (6.2)
Roofing material (n = 500)		
Aluminum sheets	278 (94.6)	16 (5.4)
Asbestos/clay/thatch	196 (95.1)	10 (4.9)
Ventilation (n = 500)		
<2 windows	74 (91.4)	7 (8.6)
2+ windows	400 (95.5)	19 (4.5)
House type (n = 500)		
Self-contained*	242 (97.2)	7 (2.8)
Single family detached	61 (93.8)	4 (6.2)
Compound	110 (94.0)	7 (6.0)
Kiosk/container/other	61 (88.4)	8 (11.6)
House age (n = 498)		
≤10 years	172 (96.1)	7 (3.9)
>10 years	151 (94.4)	9 (5.6)
Don’t know	149 (93.7)	10 (6.3)
Duration of residence (n = 494)		
≤6 months	52 (92.9)	4 (7.1)
>6 months	417 (95.2)	21 (4.8)
Occupancy at 2ppr** (n = 500)		
1-2 ppr	302 (95.6)	14 (4.4)
>2 ppr	172 (93.5)	12 (6.5)
Repair required (n = 496)		
Yes	197 (90.4)	21 (9.6)
No	273 (98.2)	5 (1.8)
House tenure (n = 499)		
Owner/caretaker	276 (94.8)	15 (5.2)
Renter	197 (94.7)	11 (5.3)
Affordability of rent (n = 497)		
Yes	121 (96.5)	5 (4.0)
No	82 (94.3)	5 (5.7)
Not applicable	269 (94.7)	15 (5.3)
Privacy (n = 500)		
Yes	403 (97.3)	11 (2.7)
No	71 (82.6)	15 (17.4)
Electricity (n = 500)		
Regular	255 (94.8)	14 (5.2)
Irregular	219 (94.8)	12 (5.2)
Primary water source (n = 500)		
Pipe/borehole	318 (94.6)	18 (5.4)
Vended/stream/rainwater	156 (95.1)	8 (4.9)
Refuse disposal system (n = 499)		
House to house by service provider	195 (92.9)	15 (7.1)
Traditional methods/communal collection	289 (96.2)	4 (3.8)
Type of sanitation facility (n = 496)		
Flush to septic tank	288 (97.3)	8 (2.7)
Pan latrine	102 (91.1)	10 (8.9)
Pit latrine/open defaecation	77 (91.7)	7 (8.3)
KVIP***	3 (75.0)	1 (25.0)
Sanitary facility in the house (n = 500)		
Present	333 (96.2)	13 (3.8)
Absent	141 (91.6)	13 (8.4)
Sanitation facility shared (n = 500)		
Yes	211 (92.5)	17 (7.5)
No	263 (96.7)	9 (3.3)
Environmental sanitation		
Satisfactory	321 (95.8)	14 (4.2)
Not satisfactory	153 (92.7)	12 (7.3)

*self-contained houses have kitchen and sanitation facilities ensuite, **ppr = persons per room, ***Kumasi ventilated pit toilet(a double chambered pit toilet).

**Table 3:** Descriptive analysis of numerical parameters in the housing survey, Greater Accra Region, Ghana, 2012

**Variable**	**Frequency**	**Range**	**Mean**	**Standard deviation**
Age	379	1-79	37.45	14.40
Occupancy (ppr)	500	1-13	2.43	1.55
Rooms per house*	499	1-30	4.37	3.14
Persons per house*	500	1-66	6.91	6.25

*Median values were 4 rooms per house and 5 persons per house respectively.

Problems with the indoor quality of the dwelling are shown in Table 4: dust particles (65.4%, n = 327) and outdoor smoke (52.8%, n = 264) were predominant. Outdoor smoke was included among problems affecting indoor quality of the house as it can filter in through open windows and doors especially in ground floor buildings. Mould growth was reported by 187 (38.0%) respondents. Three hundred and forty six (69.4%) occupants had toilet facilities in their home and 78 (15.6%) were shared facilities. Two types of sanitation facilities were commonly used: the water closet 296 (59.7%) and the pan latrine 116 (22.6%).

**Table 4:** Problems of the indoor environment in homes of study participants, Greater Accra Region, Ghana, 2012 (n = 500)

**Variables**	**Frequency (%)**
Pests	425 (85.0)
Outdoor smoke*	264 (58.8)
Animals (pets, poultry)	195 (39.0)
Indoor smoke	171 (34.2)
Foul odour	115 (23.0)
Dryness	112 (22.4)
Poor air exchange	109 (21.8)
Dampness	77 (15.4)
Wind draught	64 (12.8)

*Infiltration of the house by smoke outdoors.

### Living conditions

Two hundred and sixty nine (53.8%) respondents reported they had a constant supply of electricity and 336 (67.2%) respondents obtained water supplies from piped systems within their premises/yards and boreholes within the neighbourhood. Among those who treated their water supply, the main forms of treatment were: filtration only 102 (20.4%); filtration and boiling 80 (16.0%) and boiling only 42 (8.4%). The main methods of refuse disposal were collection in household refuse bins, 204 (40.8) and burial in a backyard pit with eventual burning, 102 (20.4%). House to house collection of refuse by private service providers was available to 210 (42.1%) houses and refuse disposal was done weekly (42.5%; n = 212). Pests were reported in 359 (71.8%) houses. Those commonly mentioned were: mosquitoes 359 (71.8%), flies 277 (55.5%), ants 196 (39.3%) and rodents 158 (31.7%). Common pest control methods used were insecticides, 282 (56.5%), mosquito coils, 194 (39.0), insecticide treated nets, 173 (34.7%) and rat traps, 102 (20.4%). Animals were kept by 195 (39.1%) respondents and they came in contact with food in 73 (14.6%) houses.

Based on a sanitation checklist, 335 (68.2%) dwellings were found to be in satisfactory condition. The checklist was not applied in 19 (3.8%) homes due to lack of consent.

Four hundred and fourteen (82.8%) occupants acknowledged their houses offered privacy and 284 (57.3%) were satisfied with the state of their houses. On the other hand, 195 (63.3%) respondents feared ejection from their houses.

### Reported health

In response to whether specific diseases were common, the following affirmative responses were obtained: malaria (51.4%, n = 257), typhoid (10.4%, n = 52), skin infections (9.4%, n = 47), respiratory tract infection (8.8%, n = 44), gastroenteritis (4.0%, n = 20) and measles (0.4%, n = 2). Two hundred and thirty seven (47.4%) respondents acknowledged that a household member had been ill with one of five diseases (malaria, typhoid, skin infections, respiratory tract infection and gastroenteritis) in the past six months. Surprisingly, when self-rated health was dichotomized, 474 (94.8%) respondents rated their health as good. Respondents were asked about ten specific physical complaints experienced at least once a month and affirmative responses obtained are shown in Table 5. At least one in four occupants reported: headaches (65.3%; n = 326), cough (64.3%; n = 320), and dizziness (25.2%; n = 126).

**Table 5:** Physical complaints reported by study participants, Greater Accra Region, Ghana, 2012 (n = 500)

**Physical complaints**	**Frequency (%)**
Headaches*	326 (65.3)
Cough**	320 (64.3)
Dizziness	126 (25.2)
Chest pain or tightness	122 (24.4)
Abdominal pain	114 (22.8)
Itchy skin	94 (18.8)
Watery eyes	83 (16.6)
Fever	71 (14.2)
Loose stools	50 (10.0)
Hearing impairment	26 (5.2)

*n = 499; **n = 498.

Table 6 shows the correlates of occupant health. Thirteen variables were associated with self-reported health: namely gender, employment status, income, ventilation, house wall material, odours, stale air, privacy, shared facilities, hand washing facility, type of house and house repair status. Only two variables were independent risk factors for poor self-rated health: occupants who lacked privacy were eight times more likely to report poor self-rated health when compared to peers who did not lack privacy [OR = 8.16, 95% CI 2.86-23.26]. Women were three times more likely than men to report poor health [OR = 2.98, 95% CI 1.06-8.35]. Location was protective [OR = 0.14, 95% CI 0.03–0.53]. Respondents living within Accra were 86% less likely to rate their health poorly.

**Table 6:** Bivariate and multivariable analyses of sample and housing characteristics and occupants’ self-reported poor health, Greater Accra Region, Ghana, 2012

***Variable***	***Self-rated occupant’s health***		***Unadjusted OR, 95% CI***	***P = Value***	***Multivariable analysis (R***^***2***^ ***= 33.8) Adjusted ORs (95% CI)***
	***Good health n(%)***	***Poor heath n(%)***			
**Sex**					
Male	245(96.8)	8(3.2)	1		1
Female	229(92.7)	18(7.3)	2.41 [1.03-5.64]	0.044	2.98 [1.06-8.35]
**Employment status**					
Formal	188(97.9)	4(2.1)	1		1
Informal	285(92.9)	22(7.1)	3.62 [1.23-10.66]	0.013	0.79 [0.63-0.99]
**Income**					
High	100(99.0)	1(1.0)	1		1
Low	374(93.7)	25(6.3)	6.68 [0.90-49.93]	0.041	[not included]
**# of windows per bedroom**					
2 or more	393(96.3)	15(3.7)	1		1
< 2 windows	81(88.0)	11(12.0)	3.56 [1.58-8.03]	0.03	1.33[0.90-1.96]
**House wall material**					
Cement	367[95.1]	19[4.9]	1		1
Non-cement	107[93.9]	7[6.1]	1.26 [0.52-3.09]	0.63	[not included]
**Odour present**					
No	370(96.1	15(3.9)	1		1
Yes	104(90.4)	11(9.6)	2.61 [1.16-5.85]	0.028	2.67 [0.68-10.43]
**Stale air present**					
No	378(96.7)	13(3.3)	1		1
Yes	96(88.1)	13(11.9)	3.94 [1.77-8.77]	0.001	3.89 [0.91-10.45]
**Has privacy**^**1 **^**in the house**					
Yes	403(97.3)	11(2.7)	1		1
No	71(82.6)	15(17.4)	7.74 [3.41-17.54]	<0.001	8.16 [2.86-23.26]
**Location**					
Outside Accra	89(89.0)	11(11.0)	1		1
Within Accra	385(96.3)	15(3.7)	0.32 [0.14-0.71]	0.009	0.14 [0.04-0.53]
**Shares toilet facility**					
No	263(96.7)	9(3.3)	1		1
Yes	211(92.5)	17(7.5)	2.35 [1.03-5.39]	0.044	1.55 [0.44-5.44]
**Hand washing facility**					
Present	226(97.0)	7(3.0)	1		1
Absent	248(92.9)	19(7.1)	2.47 [1.02-5.99]	0.044	1.03 [0.36-2.91]
**Type of house**					
Self-contained*	303(96.5)	11(3.5)	1		1
Not self-contained/other	171(91.9)	15(8.1)	2.42 [1.09-5.38]	0.036	0.804 [0.25-2.57]
**House needs repair**					
No	277(98.2)	5(1.8)	1		1
Yes	197(90.4)	21(9.6)	5.91 [2.19-15.91]	<0.001	1.877 [0.68-5.91]

Note: Significance considered at p-value < 0.05 and if the OR estimate did not cross the null for 95% CI. Fishers Exact Test used for small cell counts. *Self-contained – means house has an inbuilt sanitary and kitchen facilities.

^1^Privacy in a house–response to the question: ‘Do you have privacy at home?’

The final model included: type of house, house wall material, shared toilet, hand washing facility, number of windows per bedroom, sex, employment status, household income, stale air present, odour present, house needs repair, has privacy in the house, and location.

## Discussion

Results from the present survey of five hundred respondents aged 18 years or older in five neighbourhoods (four urban and one rural), situated in the Greater Accra Region of Ghana, lend support to earlier studies in Ghana [13,14] and a recent survey reported in the Ghana Housing profile [19]. These surveys draw attention to housing quality as a major determinant of health.

The present study indicates that most residents in the Greater Accra region are living within the threshold occupancy limit. Similar to the Ghana Living Standards Survey (GLSS5) which reports mean room occupancy of 2.3 persons per room (ppr) in urban Ghana, 2.1 ppr for Accra and 2.4 ppr for other urban areas, the present survey found a mean room occupancy of 2.43 ppr with three in five persons living within this limit. However, it is worrisome that two in five persons live outside this threshold in crowded houses. Crowding has been associated with increased rates of infectious transmission, poor mental health, short stature and stomach cancer [13].

**Figure 2:**
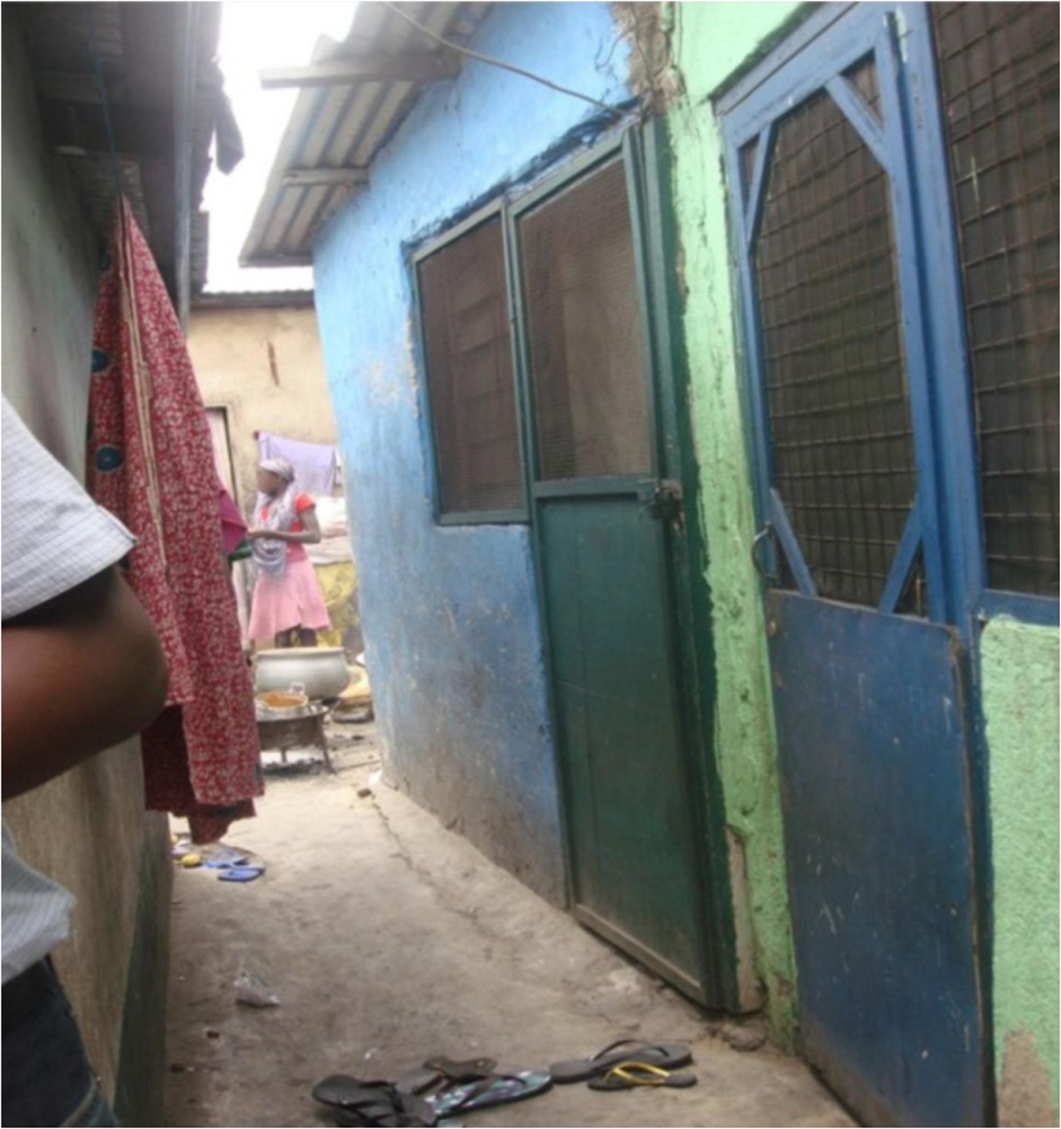
**A multi-tenement structure in an informal settlement.** Single windows on only one side of the building result in poor ventilation. Single rooms are occupied by families and lack privacy.

The Ghana housing profile reports that corrugated aluminium sheets were the main roofing material used by the majority of households as we also found in this survey, (68% v. 59%). Likewise, buildings were mostly made from cement blocks as also reported for urban Ghana (77% v. 76%) [19]. Twenty three per cent of respondents lived in compound houses which are often family houses and believed to promote communal living. Extensions of these are built and given out for rent by rooms resulting in higher number of rooms per house. Our results indicate that the number of rooms ranged from one to thirty with a mean value of four rooms per house. Compound houses and multi-tenement buildings (see Figure 2) which have a higher number of rooms and occupants also have the drawback of lacking privacy [19]. The results show that privacy was associated with poor self-rated health and those who lacked privacy were eight times more likely to report poor health compared to their peers. This suggests that structures which enhance privacy should be a focus for future housing units. This is increasingly depicted in the developing gated communities in Accra, although the cost of acquiring such houses makes them less accessible to the urban poor.

**Figure 3:**
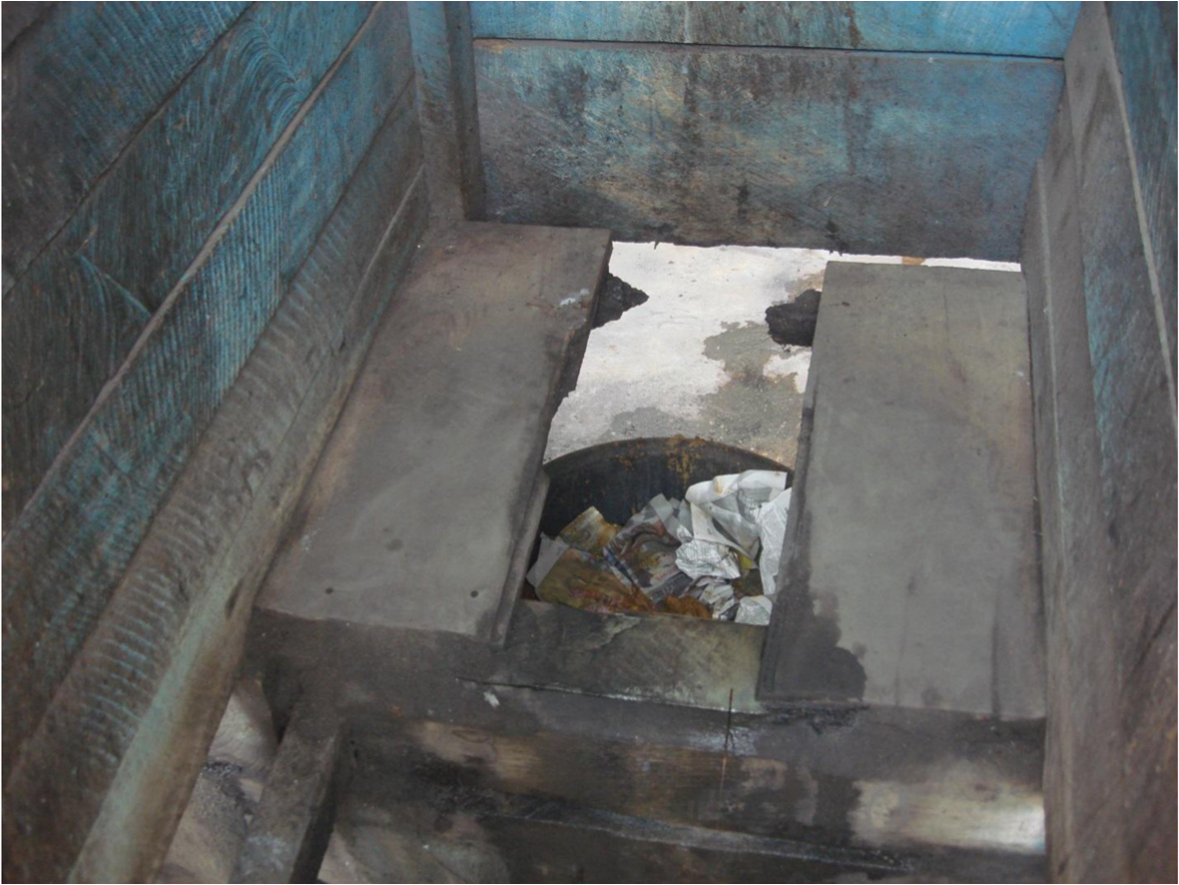
**A bucket latrine: banned but still in existence.** The latrine is a public toilet in one of the neighbourhoods.

**Figure 4:**
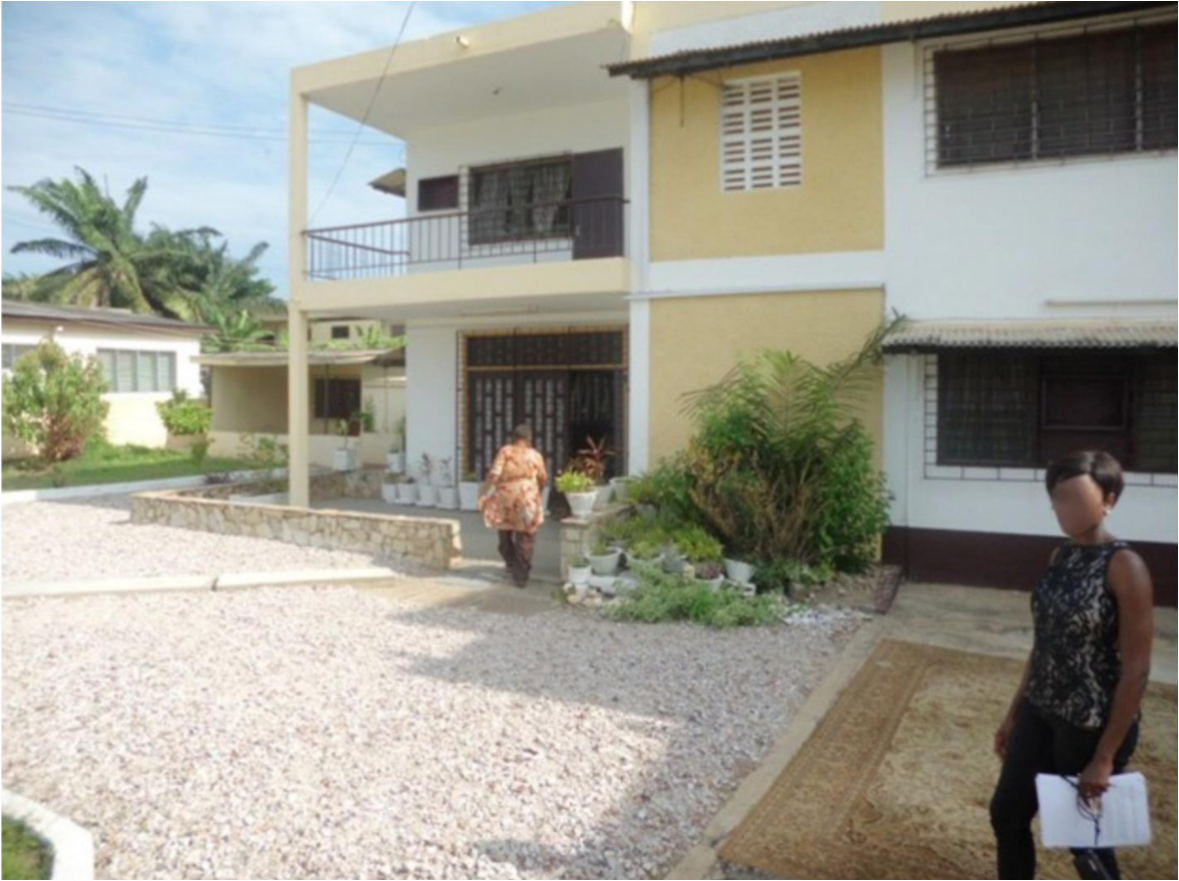
**A duplex in a middle income neighbourhood.** A family house in a neat, well-kept compound in one of the urban neighbourhoods.

The Millennium Development Goals (MDGs) and WHO/UNICEF Joint Monitoring Programme (JUMP) for Water Supply and Sanitation have classified a sanitary facility as unimproved if it is otherwise acceptable (flush or pour flush to a piped sewer system, septic tank or latrine; ventilated improved pit latrine, pit latrine with slab and composting toilet) but shared between two or more households or if a public toilet is used [20]. In a previous survey in Accra, the dominant types of toilet facilities found were the ventilated improved pit latrine (VIP) and flush to septic tank toilets used by 42% and 26% of respondents respectively [20]. In the present survey, the corresponding proportions were 16% and 60%. Although the flush toilets were dominant, the next most common was the pan latrine used by 23% of the respondents. This form of latrine was outlawed in Ghana in the last decade but survey results indicate they are still existent (see Figure 3). This type of sanitation facility is often shared where it exists and it is unacceptable because it permits human contact with human excrement. Although 69% of houses had a sanitation facility in the dwelling, nearly half of them were shared (46%), making them unimproved sanitation facilities. The MDG target 7 calls for 54% of the population to have access to improved sanitation by 2015 [25]. The high proportion of shared sanitation facilities in present survey included four neighbourhoods in the national capital where access to amenities is considered to be better than other parts of the country. It can be assumed that the situation would be worse in other parts of the country but other regional surveys are required to prove this. The effect of shared sanitation facilities was affected by other factors assessed in the survey as it was not independently associated with poor self-rated health. Location within Accra was associated with good self-rated occupant health. Living within the capital confers greater access to basic amenities, health care and other factors which promote health. People who lived within Accra (see Figure 4) were 86% less likely to report poor self-rated health compared to those who lived outside Accra. The location of slum neighbourhoods in central neighbourhoods in Accra which offers relative ease of access to amenities and health facilities was also reported by Fink et al. [26].

**Figure 5:**
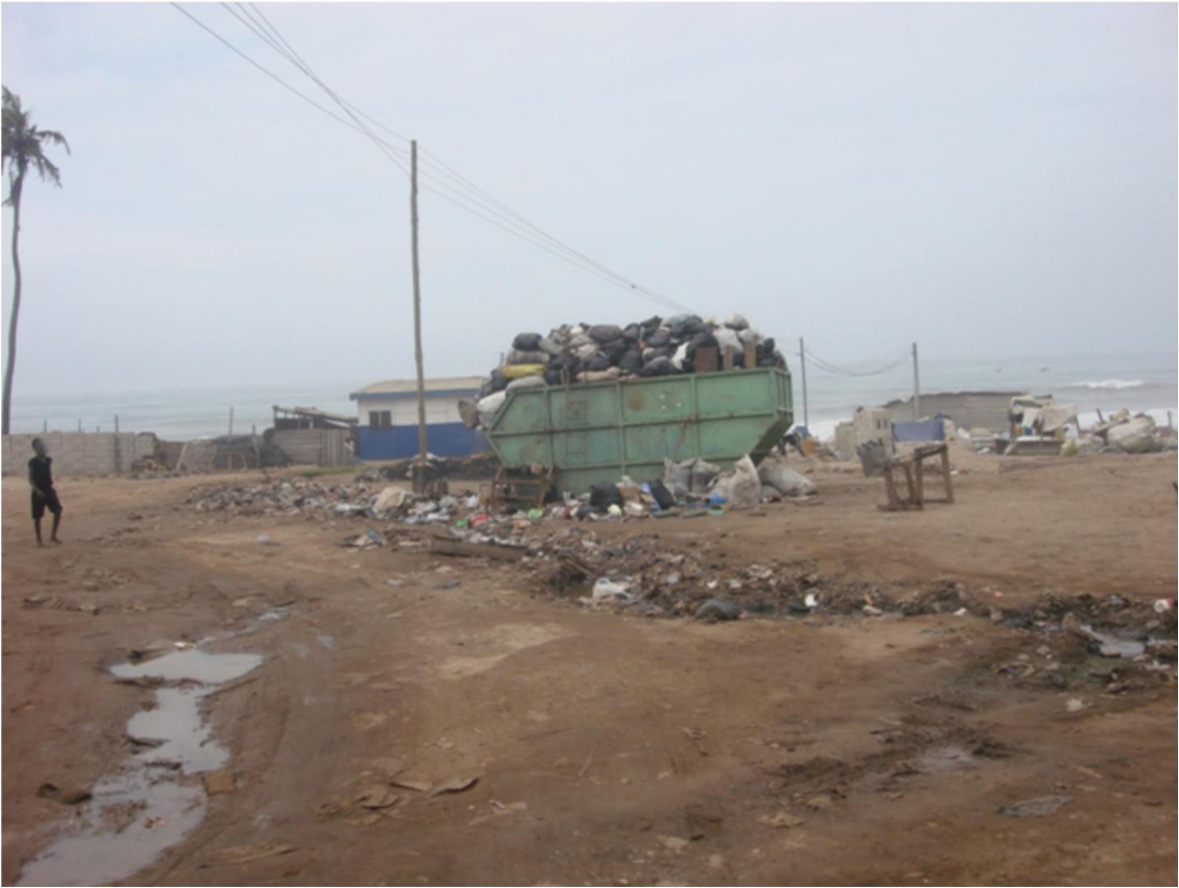
**A skip at a communal collection point for refuse disposal in one of the neighbourhoods.** The skip is located a short distance from residential dwellings. Wind draughts from the beach blow in malodorous air towards the dwellings.

The presence of uncollected refuse, litter and refuse dumps, serve as harbourage for vermin, rodents and vectors (refer Figure 5). Refuse collection by a private service provider was reported in two of every five houses in the survey which is inadequate and offers the opportunity for refuse to accumulate in drainages and dumps. Uncollected refuse also constitutes an aesthetic nuisance in addition to unacceptable odours emitted from decaying materials. The presence of wind draughts in such areas facilitates the dispersion of bio-aerosols from mould on moist refuse and dust particles which may serve as vehicles for other infectious material. It also causes a stench within the environment. Although the presence of odours was significantly associated with poor self-rated health, this effect was not independent of other factors investigated in this survey. In spite of this limitation, the public health importance of a clean and healthy environment is overriding and it is recommended that environmental quality in terms of sanitation and waste disposal should be integrated with improvements in housing. In the health sector, Environmental Health Officers will require adequate legal backing to enable them address nuisances effectively without undue interference. Monitoring mechanisms for housing quality other than periodic demographic and health surveys can offer hypothetical explanations for disease profiles observed in local health facilities. In turn, such reports alert relevant authorities to situations requiring further investigation and priority attention for remediation and prevention of potential outbreaks.

**Figure 6:**
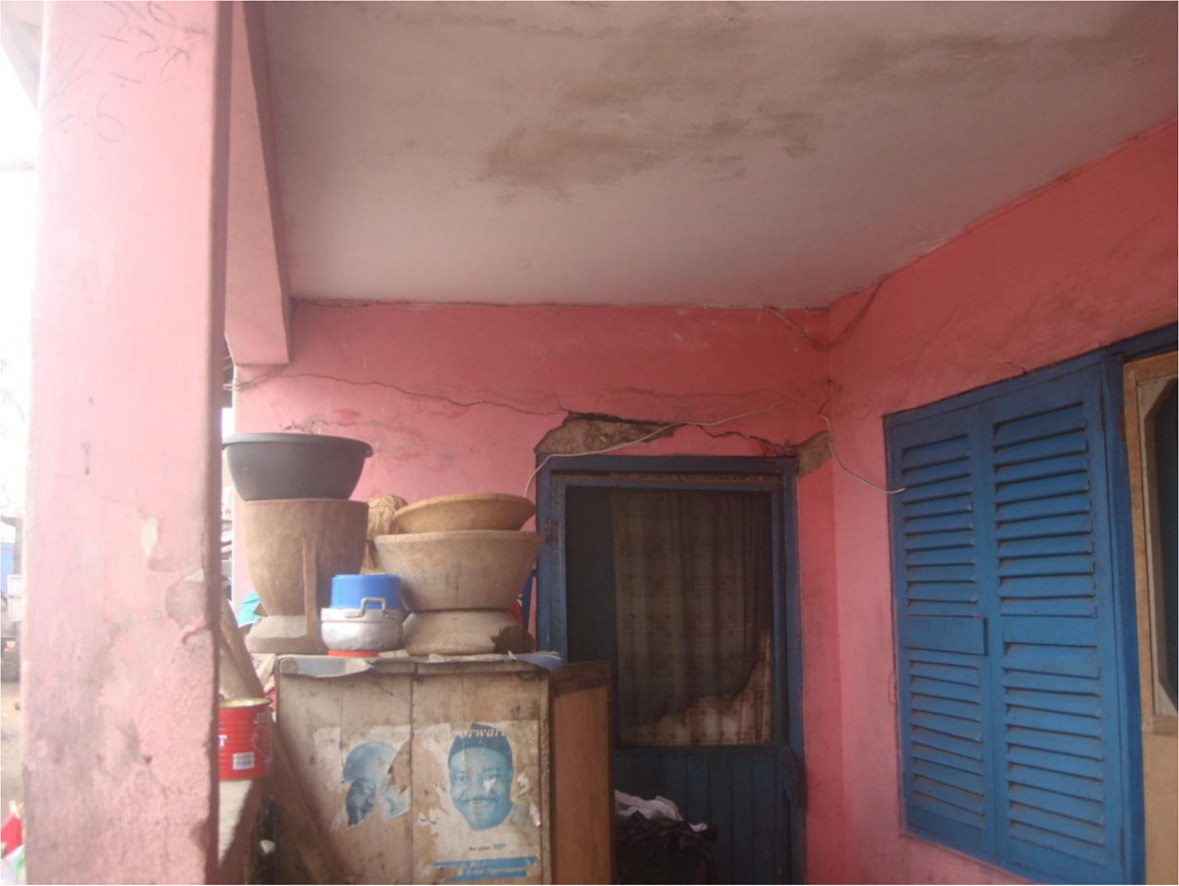
**One of the houses in poor repair status.** The house has cracks in the walls, a torn door net, and electrical wiring hanging off the walls.

In the present survey, 47% of respondents admitted to have a household member that was affected by common illnesses and between 17% and 65% reported physical complaints known to be associated with poor housing quality (see Figure 6). It is surprising that despite poor environmental quality and a high prevalence of illness in the surveyed neighbourhoods, only 5% of respondents rated their health as poor. This may be related to the short duration of some of the illnesses assessed. Since four of the five neighbourhoods were urban areas with relatively better access to health care facilities and basic amenities, it is possible that restoration to health was swift or illness was less debilitating. Consequently, respondents had a positive view of health especially if they were not sick at the time of interview or if illness did not limit physical or economic activity. Although health outcomes were different, Fink et al. [26] noted a similar positive view of health among respondents [26]. In a study investigating slum residence and health among adult women in Accra, the authors found positive health outcomes and suggested a self-selection theory where women found in informal settlements are more driven, optimistic and tend to have a more positive view of their health [26]. They also suggested that because these women were generally in stronger health, environmental factors tend to have less impact on health [26]. The difference in health outcomes between the two studies may be due to different methods employed in assessing health outcomes.

The effect of gender remained significant in the study and may be explained by the fact that generally women tend to spend more time in the domestic environment than males by sheer reason of their housekeeping roles. A similar finding was reported in the study by Arku et al., [13]. The implications for health deserve emphasis as women are primary caregivers in the family and they need to be healthy to play their role effectively.

The government of Ghana has demonstrated commitment to ensure an improved housing stock in the near future. One such example was the proposed public private partnership between the Government of Ghana and STX Engineering and Construction, Ghana Limited, for the development of 200,000 units of affordable housing for the low and middle income group known as the Ghana Housing Project, with additional 300 units for senior state officers over five years. This was later aborted due to unresolved internal conflicts. Otherwise this may have been a laudable project as the gated communities offered by private developers serves the high end of the housing market demand. Notwithstanding, the Government of Ghana continues to encourage private developers to provide low income housing by offering incentives such as land and removal of duties from imported construction materials [19]. However, the pace of estate development hardly matches the demand for housing. Rather up to 90% of all housing is provided by individual householders who contract private builders to build for them at a pace dictated mainly by availability of funds and cost of building materials which ultimately takes many years. The estimated need for about two million self-contained dwellings (at one per household) by 2020 in Ghana remains a challenge under the circumstances. The rights to privacy and health through adequate housing remain issues for public health action.

## Conclusions

● The association between self-reported occupant health and residential characteristics was found to be evident among females and in occupants of houses characterized by lack of privacy.

● Despite the prevalence of common neighbourhood illnesses and physical complaints, occupants in the surveyed neighbourhoods tended to report good self-rated health.

● Housing improvement which enhances privacy has the potential to promote self-rated health and should be advanced as an issue for public health action.

## Competing interest

The authors declare that they have no competing interests.

## Authors’ contributions

EAU conceived the study design, performed the statistical analysis, and drafted the manuscript. KAA participated in the design and coordination of the study. AEY participated in the study design and drafting of the manuscript. FMB participated in the statistical analysis and drafting of the manuscript. All authors read and approved the final manuscript.

## Pre-publication history

The pre-publication history for this paper can be accessed here:

http://www.biomedcentral.com/1471-2458/14/244/prepub
